# A Content Analysis and Population Exposure Estimate Of Guinness Branded Alcohol Marketing During the 2019 Guinness Six Nations

**DOI:** 10.1093/alcalc/agab039

**Published:** 2021-06-03

**Authors:** Alexander B Barker, Jaspreet Bal, Rachael L Murray

**Affiliations:** Division of Epidemiology and Public Health, University of Nottingham, Clinical Sciences Building, City Hospital Campus, Hucknall Road, Nottingham NG5 1PB, UK; SPECTRUM Consortium, UK; Division of Epidemiology and Public Health, University of Nottingham, Clinical Sciences Building, City Hospital Campus, Hucknall Road, Nottingham NG5 1PB, UK; SPECTRUM Consortium, UK; Division of Epidemiology and Public Health, University of Nottingham, Clinical Sciences Building, City Hospital Campus, Hucknall Road, Nottingham NG5 1PB, UK; SPECTRUM Consortium, UK

## Abstract

**Aims:**

To quantify Guinness-related branding in the 2019 Guinness Six Nations Championship.

**Methods:**

Content analysis of Guinness-related branding (‘Guinness’ and the alibi brand ‘Greatness’) was shown during active play throughout all 15 games of the 2019 Guinness Six Nations Championship. The duration of each appearance was timed to the nearest second to provide information on the amount of time that Guinness-related branding was shown on screen. Census data and viewing figures were used to estimate gross and per capita alcohol impressions.

**Results:**

Our coding identified a total of 3719 appearances of two logos of which 3415 (92%) were for ‘Guinness’ and 304 (8%) were for ‘Greatness’. ‘Guinness’ imagery was present for 13,640 s (227.3 min or 3.8 h, 16% of total active play time), ‘Greatness’ was present for 944 s (15.7 min, 1% of total active play time), with a combined total of 14,584 s across all games (243 min or 4.05 h, 17% of active play time). The 15 games delivered an estimated 122.4 billion Guinness-related branded impressions to the UK population, including 758 million to children aged under 16.

**Conclusions:**

Alcohol marketing was highly prevalent during the 2019 Guinness Six Nations Championship and was a significant source of exposure to alcohol marketing and advertising for children, likely influencing youth alcohol experimentation and uptake.

## INTRODUCTION

Alcohol consumption in England caused 5698 alcohol-specific deaths in 2018 (Office for National Statistics, 2020) as well as further morbidity from serious health conditions, such as stroke, heart attack and cancer (NHS, 2020). Furthermore, an estimated 602,000 people in England currently suffer from alcohol dependence (Public Health England, 2021). The morbidity and mortality associated with alcohol consumption, including tangible, direct costs (such as health, justice and welfare systems), indirect costs (such as absenteeism, unemployment, decreased output or lost working years due to premature pension or death) and intangible harms (such as pain or suffering), result in an economic burden of between £21 and £52 billion each year (Public Health England, 2016), and this is clearly a public health priority.

Exposure to advertising or other audio-visual alcohol content (AVC) in the media is associated with alcohol initiation and subsequent use by adolescents (Anderson *et al.*, 2009; Smith and Foxcroft, 2009; Hanewinkel *et al.*, 2014; Chang *et al.*, 2016). Televised sporting events are popular with young people, and research has shown that young people under the age of 16 are exposed to alcohol branding which occurs at the venue and is broadcast on television, such as Carlsberg branding in the Euro 2016 football Championship (Murray *et al.*, 2018) or Heineken in the F1 motorsports championship (Barker *et al.*, 2020b). A systematic review of the effects of alcohol sports sponsorship has shown a positive association between the exposure to alcohol sports sponsorship and self-reported alcohol consumption, including in secondary school-aged children (Brown, 2016). While commercial advertising of alcohol and alcohol content in broadcast programmes are regulated in the UK to prevent adolescent exposure, the Advertising Standards Authority commercial advertising regulatory codes do not cover broadcast footage of imagery arising from sporting events (Advertising Standards Authority, 2019a, 2019b). Such footage is considered programme content and therefore in theory should be covered by Ofcom regulations (Ofcom, 2017), however, Ofcom has no remit over sports sponsorship deals (Ofcom, 2016; Ingram, 2018). Additionally, alcohol sports sponsorship is self-regulated by the Portman Group, a group composed of alcoholic beverage producers, including Guinness. The Portman Group Sports Sponsorship code states that it seeks to ensure that alcohol is promoted in a socially responsible manner and only to those over 18 and further stipulates that drinks companies must use reasonable endeavours to ensure that at least the aggregate of 75% of the audience or spectator profile are aged over 18 (Portman Group, 2014). However, while 75% of an audience may be adults, televised sports programmes are still popular with children and young people (Ofcom, 2019), and young people and adolescents are regularly exposed to this content (Critchlow *et al.*, 2019). Alcohol advertising during televised sporting events is thus a potentially unregulated source of exposure to alcohol content in the UK (Barker *et al.*, 2020b), which is particularly pertinent for young people.

Guinness was announced as the title partner for the Six Nations Rugby Championship in December 2018 (Six Nations Rugby, 2018), allowing Guinness to build on its ‘already strong presence in and around the Home Nations stadia’ (Six Nations Rugby, 2020). Furthermore, in countries where alcohol marketing is prohibited, such as France due to the Loi Evin (LegiFrance, 2021), alcohol marketing has adopted alibi marketing practices (whereby core elements of a brand’s identity, such as a strapline, word, colour or shape, are used in advertising instead of the brand’s name or logo) to bypass regulation (Murray *et al.*, 2018). In a similar way to Carlsberg using the tagline ‘Probably’ in the Euro 2016 Football Championship (Murray *et al.*, 2018) and Philip Morris’ Marlboro alibi brand ‘Mission Winnow’ in the Formula 1 Championship (Barker *et al.*, 2020b), in the 2019 Six Nations Rugby Championship, Guinness used the alibi brand ‘Greatness’ (in the same font and colour as the Guinness brand) to market its products. As such, we present a content analysis of Guinness-related branding for the entire 2019 Six Nations tournament and estimate population exposure to this content.

## METHODS

We descriptively studied alcohol content and estimated exposure to the entire 2019 Six Nations Championship. Live coverage of all matches was recorded in entirety. Our coding, which adapted from methods used in a previous study (Murray *et al.*, 2018) included any time of active play in the game from the kick-off whistle in the first and second halves to half-time or the final whistle, respectively. Our coding instrument separately listed each appearance of ‘Guinness’ and the alibi brand ‘Greatness’ (which was displayed in the characteristic white font with black background). For each appearance, the time started and time ended in minutes and seconds by the match period were recorded. Audio-visual occurrences that appeared uninterrupted were recorded; partially visible brands were not counted. The duration of each appearance was timed to the nearest second to provide information on the amount of time that Guinness-related branding was shown on the screen. All information was recorded in a separate excel file before being transferred to SPSS v.24 for analysis. To ensure accuracy and reliability in the coding method, 1 of the 15 games (7%) was coded independently by two coders with any differences resolved by discussion; the level of agreement between the two coders was 95%.

To estimate the UK population exposure to Guinness-related content, we estimated UK audience exposure using viewing data from Digital.I (Digital.I, 2018) and UK mid-year population estimates for 2018 (Office for National Statistics, 2019) combined with the numbers of alcohol appearances to estimate gross (the total number of impressions delivered to the UK population) and per capita (the number of impressions delivered to each person), as has been previously reported (Barker *et al.*, 2018, 2019, 2020a, 2020b, 2020c, 2020d; Murray *et al.*, 2018). The method involves combining viewership (obtained from viewing figures) with the number of appearances per game to calculate gross impressions as the estimated number of exposures delivered. Dividing gross impressions by population mid-year estimates provided per capita impressions, the estimated number of alcohol impressions delivered to each person. Both gross and per capita impressions were computed by age group.

## RESULTS

### Content analysis

The 15 rugby matches studied were transmitted between 1 February and 16 March 2019. Eight of the matches were shown on BBC1 and seven matches were shown on ITV. The broadcasts included a total of 87,480 s (1458 min) of active play.

Our coding identified a total of 3719 appearances of the two logos of which 3415 (92%) were for ‘Guinness’ and 304 (8%) were for ‘Greatness’. ‘Guinness’ imagery was present for 13,640 s (227.3 min or 3.8 h, 16% of total active play time), ‘Greatness’ for 944 s (15.7 min, 1% of total active play time), with a combined total of 14,584 s across all games (243 min or 4.05 h, 17% of active play time).

Branding appearances were seen in a number of sources, including the sideline, superimposed onto the centre of the pitch, flag posts, sponsor walls (behind close-up shots of team members) goal posts, stewards’ uniforms or billboards on display in the seating areas in the stadium. The largest category of appearances occurred through the branding being superimposed onto the centre of the pitch, accounting for 1459 appearances (42.7% of appearances).

### Population exposure

We estimate that 15 matches delivered 122.4 billion Guinness-related branded impressions (95% confidence interval (CI): 112.4–131.8) to the UK population, including 758 million (95% CI: 660–860) to children aged under 16 (see Supplementary Table S1).

## DISCUSSION

The current study shows that alcohol marketing was highly prevalent during the 2019 Guinness Six Nations Championship which was seen by millions of viewers and that it included more than 758 million gross branded impressions to children under the age of 16. Our study thus provides evidence that UK broadcast footage of the Guinness Six Nations Championship is a significant source of exposure to alcohol marketing and advertising for children. As there is now strong, causal, evidence to suggest that exposure to alcohol imagery in the media increases subsequent alcohol consumption (Sargent *et al.*, 2006; Anderson *et al.*, 2009; Smith and Foxcroft, 2009; Hanewinkel *et al.*, 2012; Hanewinkel *et al.*, 2014; Brown, 2016; Sargent and Babor, 2020), it is likely that the amount of alcohol marketing broadcast throughout the Guinness Six Nations has an effect on youth alcohol experimentation and uptake.

We have previously identified that alcohol advertising during televised sporting events is a potentially unregulated source of exposure to alcohol content for young people under age 16 (Barker *et al.*, 2020b), and the current study provides further evidence that this potentially unregulated advertising is widely seen by young audiences. Restrictions on, and enforcement of, alcohol advertising during sporting events are needed to protect children and adolescents from this avenue of alcohol advertising.

The Portman Group Sponsorship code states that it seeks to ensure that alcohol is promoted in a socially responsible manner and only to those aged over18. The current study shows that the 2019 Guinness Six Nations Championship delivered an estimated 758 million Guinness branded impressions to children under 16 in the UK. The Portman Group Sponsorship code therefore did not ensure that this marketing was socially responsible in protecting the under-18s. Televised sporting events are currently unregulated and an independent regulatory body, such as Ofcom, is needed to protect young people from alcohol sports sponsorship marketing content.

While there is scope under Ofcom’s powers to regulate the content in programmes broadcast in the UK, Ofcom currently has no remit over sports sponsorship deals, and these are therefore a potentially unregulated source of exposure to alcohol content for young people. Furthermore, the Guinness Six Nations Championship matches are broadcast internationally, which poses cross-border challenges for regulation. The Framework Convention for Tobacco Control (World Health Organization, 2005) is a treaty negotiated under the auspices of the World Health Organization, which provides a regulatory strategy to address addictive substances and has placed an international, comprehensive ban on the advertising, promotion and sponsorship of tobacco products (World Health Organisation, 2005). A similar comprehensive ban on the advertising, promotion and sponsorship of alcohol products would prevent young people from being exposed to this currently unregulated alcohol promotion.

In the UK, broadcasting rights to the Guinness Six Nations were shared by the British broadcasting corporation (BBC) and independent television (ITV). ITV showed commercial advertisements during their programming. While conducting this study, we recorded five advertisements for Guinness. The advertisement used for Guinness contained a unique piece of music (The Tornados—Jungle Fever) which was not popular at the time, having been released in 1962. This music was heard in the stadium three times through the television footage before and after games and was likely heard by fans in the stadium. We argue that this music acts as an audio cue associated with Guinness to influence purchasing behaviour as an adjunct or alternative to visual cues. This form of advertising has not been widely studied but should be monitored in future sporting events to establish the extent of such an approach.

Alcohol marketing for Guinness was prevalent in all matches, even in a country where alcohol marketing is prohibited. While France has statutory legislation, the Loi Evin (LegiFrance, 2021), prohibiting alcohol marketing and advertising by placing a total ban on the direct or indirect advertising of all alcoholic beverages over 1.2% ABV on television and prohibiting sponsorship of sports events by alcohol companies (Institute of Alcohol Studies, 2019), the alibi brand ‘Greatness’, which shares the same font and colour scheme as the Guinness Brand, was used in matches which took place in France. This is similar to alibi branding which has been used in this country for previous sporting events (Murray *et al.*, 2018).

All of the six nations matches were broadcast on the weekend before the 9 p.m. watershed, a time when children are likely to be watching either through personal choice or an indirect consequence of parental viewing (Jago *et al.*, 2014). The Ofcom Broadcasting Code (Ofcom, 2017) considers factors that determine whether a programme should be shown, including ‘the likely number and age range of children watching, taking into account school time, weekends and holidays‘ (Channel 4, 2017); the current study has demonstrated that footage of the Guinness Six Nations matches was broadcast at a time when likely to be seen by children and, as the current study shows, was widely seen by children and delivered millions of branded alcohol content impressions to children.

While the current study found that alcohol branding was highly prevalent in the broadcast footage of the 2019 Guinness Six Nations, we currently do not know whether this led to increased alcohol sales. Future studies should explore sales data to identify if the presence of branding translates to increased sales. The current study is limited by having only explored branding shown during active play; however, we note that branding and alcohol content may have also been prevalent during the match lead-up and halftime discussion and thus actual exposure is potentially higher. Future studies should explore this additional content to estimate the true scale of the issue. Furthermore, we also note that since the Guinness Six Nations is a European competition and that these matches can be viewed either freely or via subscription in other countries, the UK population exposure figures used in this study likely represent a small proportion of the true exposure to this content. Despite these limitations, this is the first study of its kind to explore the entire Guinness Six Nations Championship and estimate population exposure to this content.

## Supplementary Material

6_Nations_supplementary_1_agab039

## Data Availability

The datasets used and/or analysed during the current study are available from the corresponding author on reasonable request.
