# Supplementary material for: A Content Analysis and Population Exposure Estimate Of Guinness Branded Alcohol Marketing During the 2019 Guinness Six Nations
Source: Alcohol Alcohol. 2021 Jun 3;56(5):617–20. doi: 10.1093/alcalc/agab039 (PMC8521759; doi:10.1093/alcalc/agab039)
Supplement: 6_Nations_supplementary_1_agab039 [file 6_nations_supplementary_1_agab039.docx]

Table S1: Estimated Guinness related audio-visual content exposure per match

|  |  |  | Match | Scotland Vs Italy | Ireland Vs England | Scotland Vs Ireland | Italy Vs Wales | England Vs France | Wales Vs England | Italy Vs Ireland | Scotland Vs Wales | England Vs Italy | Ireland Vs France | Italy Vs France | Wales Vs Ireland | England Vs Scotland |  |  | France Vs Wales | Scotland Vs France | Total |
| --- | --- | --- | --- | --- | --- | --- | --- | --- | --- | --- | --- | --- | --- | --- | --- | --- | --- | --- | --- | --- | --- |
| Content Analysis | Guinness |  | Active Play Time (Seconds) | 711 | 719 | 793 | 1270 | 1301 | 915 | 1045 | 672 | 1290 | 920 | 1574 | 1182 | 1248 |  | Greatness (Games played in France) | 461 | 483 | 14584 |
|  |  |  |  |  |  |  |  |  |  |  |  |  |  |  |  |  |  |  |  |  |  |
| Estimated Population Exposure |  | Total | Proportion viewership (000s) | 0.049 | 0.055 | 0.066 | 0.031 | 0.052 | 0.109 | 0.019 | 0.055 | 0.037 | 0.025 | 0.017 | 0.075 | 0.043 |  |  | 0.074 | 0.051 |  |
|  |  |  | Gross Impressions (Million) | 6676.74 | 9543.02 | 10147.99 | 8461.81 | 8705.03 | 19454.23 | 5497.87 | 6267.04 | 5702.07 | 3907.22 | 4309.73 | 13340.56 | 7555.44 |  |  | 8362.37 | 4539.65 | 122470.77 |
|  |  |  | Per Capita Impressions | 10.05 | 14.37 | 15.28 | 12.74 | 13.11 | 29.30 | 8.28 | 9.44 | 8.59 | 5.88 | 6.49 | 20.09 | 11.38 |  |  | 12.59 | 6.84 |  |
|  |  |  |  |  |  |  |  |  |  |  |  |  |  |  |  |  |  |  |  |  |  |
|  |  | Under 16’s | Proportion Viewership (000s) | 0.018 | 0.022 | 0.021 | 0.008 | 0.015 | 0.040 | 0.008 | 0.015 | 0.011 | 0.006 | 0.004 | 0.027 | 0.015 |  |  | 0.019 | 0.014 |  |
|  |  |  | Gross Impressions (Million) | 46.88 | 72.11 | 61.96 | 42.86 | 46.68 | 134.83 | 44.36 | 33.54 | 31.54 | 18.42 | 21.72 | 90.58 | 48.54 |  |  | 40.13 | 23.99 | 758.14 |
|  |  |  | Per Capita Impressions | 3.72 | 5.72 | 4.92 | 3.40 | 3.70 | 10.70 | 3.52 | 2.66 | 2.50 | 1.46 | 1.72 | 7.19 | 3.85 |  |  | 3.19 | 1.90 |  |
